# The knowledge-to-action process model for knowledge translation in oral care in South Africa

**DOI:** 10.4102/sajcd.v70i1.951

**Published:** 2023-07-31

**Authors:** Jaishika Seedat

**Affiliations:** 1 Department of Speech Pathology and Audiology, Faculty of Humanities, University of the Witwatersrand, Johannesburg, South Africa

**Keywords:** knowledge-to-action process model, oral care, nursing, acute, South Africa

## Abstract

**Background:**

Literature supports the mismatch between empirical evidence and service delivery. Given this knowledge gap, it is important that research undertaken has a theoretical foundation, considers the context and stakeholders to confirm its need and that it can be feasibly implemented and sustained.

**Objectives:**

The study aimed to facilitate knowledge translation in oral care using the knowledge-to-action (K2A) process model among nurses.

**Method:**

The study was completed in an acute hospital in South Africa. A qualitative design with ethnography incorporating video-recordings and semi-structured interviews were used. A total of 139 nurses were recruited using random purposive sampling and received training on oral care, which was monitored. Inductive thematic analysis was completed.

**Results:**

The model facilitated information transfer and implementation of oral care by nurses.

**Conclusion:**

With clear directions for use and theoretical underpinning, the K2A model was well-suited to the needs of the study and stakeholders, and the complexity of the context. For challenging contexts such as public healthcare institutions in South Africa, this was ideal and critical to the success of the intervention.

**Contribution:**

When nursing managers show interest and recognise nurses for their role in implementation of interventions, the buy-in, support and sustained use of that intervention is enhanced. A model such as the K2A promotes involvement of all stakeholders (e.g. nurses and their managers) in the design and planning for implementation of an intervention, and these are critical for the successful and feasible use of the intervention.

## Introduction

Knowledge translation provides a means of merging existing knowledge and new evidence to close the gap between research and practice. Barriers to knowledge translation in South Africa (SA) and other similar contexts, range from being structural and resource related, to systemic rules and regulations as well as human traits of attitudes, perceptions, and assumptions (Grimshaw et al., 2012). Ultimately, contextual considerations are fundamental to any change in healthcare service delivery. Internationally, healthcare practitioners struggle to translate empirical evidence into intervention while facilitating optimal and timeous outcomes (Field et al., 2014). The mismatch that exists between research findings and implementation thereof confirms that change is not automatic and if it happens, sustainability must not be assumed. The National Health Service Institute for Innovation and Improvement has estimated that up to 70% of all organisational change is not sustained over time (Ilott et al., 2013a). Selecting an appropriate framework, theory or model would provide the necessary interface to address the diverse and dynamic variables that hinder knowledge translation (Damschroder et al., 2009) and once selected should elucidate and ensure sustainability of the intervention with measures to monitor the variables that are likely to influence the intervention over time (Chambers et al., 2013). This study reveals how the knowledge-to-action (K2A) framework devised by Graham and colleagues (2006), facilitated implementation of oral care among nurses working in an acute hospital in SA.

**FIGURE 1:**
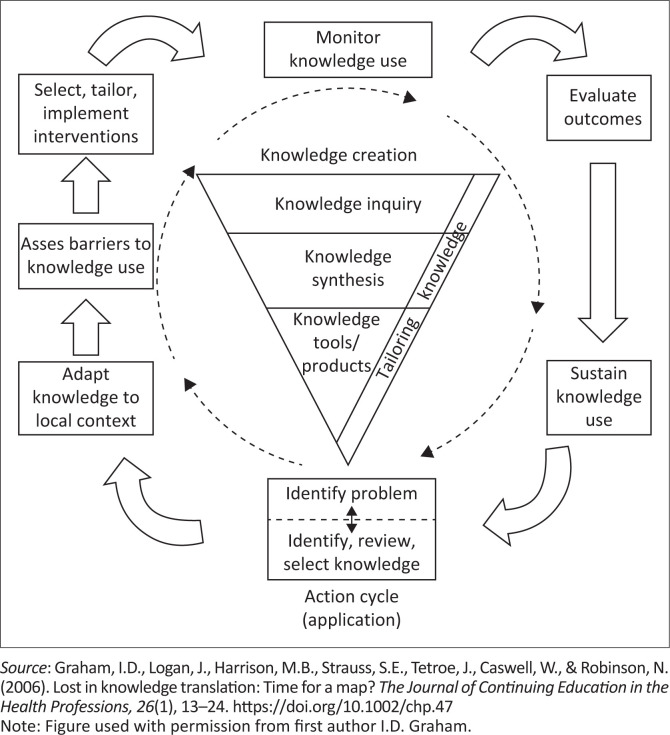
The knowledge-to-action (K2A) process model.

Following a review of the literature, different models to facilitate implementation of research, that is, knowledge translation have been described in the literature (Davis & Conti, 2003). Insufficient attention given to the patient in the model by Pathman and colleagues (1996) and the minimisation of context in the model by Green and colleagues (1980) made both these models unsuitable for the requirements of this study. Variables such as persons involved in the intervention (e.g., nurses), the recipients of the intervention (i.e., the patient), as well as the systemic parameters operating within the context (e.g., a hospital) cannot be divorced from the how, who or why of the actual process or intervention (i.e., oral care) that requires changing. Graham et al. (2006) conceptualised the K2A model as a synthesis of available evidence on knowledge translation (Figure 1). This model was applicable for this study context in the sense that the process of knowledge creation is dynamic and starts from what is known. Of relevance is the focus for knowledge to be adapted to the needs and circumstances of the local context, thereby addressing feasibility of the intervention for the context. The K2A model offered an inclusive framework to consider the variables that contribute to the challenges that nurses faced working in public healthcare in SA.

Graham et al. (2006) suggest that knowledge is derived from an empirical and experiential basis. As there is movement of knowledge through the funnel, it becomes more distilled and refined (Graham et al., 2006, p. 18). Knowledge inquiry is first generation knowledge that is in its natural state and largely unrefined. Knowledge synthesis represents the aggregation of existing knowledge. This process involves feedback, discussion and critique to understand the needs of the stakeholders and context to formulate the research questions and aims of the planned intervention. The third tier represents knowledge tools or products. The purpose of the tools is to present knowledge in a clear, concise and user friendly way with explicit meanings to facilitate uptake and application of knowledge (Graham et al., 2006). The action cycle frames the knowledge creation funnel and represents the activities that are necessary to apply the knowledge. Graham et al. (2006) recommend the identification of facilitators or supports that may prove beneficial in implementation of the intervention. The next phase of the model represents the dissemination, planning and execution of the intervention to facilitate and promote awareness and implementation of knowledge. Identifying barriers and facilitators is crucial for this phase to proceed effectively. Once the implementation initiatives commence monitoring the use of knowledge begins and proceeds into the next phase. For this, Graham et al. (2006) notices the importance of defining what constitutes knowledge use to allow measurement. It is important to monitor knowledge use to assess the extent to which it is translated into action. The role of the intervention in bringing about the desired change is also evaluated at this phase. It is thereafter necessary to define the impact of using the knowledge in the next phase, that is, whether the efforts to improve the uptake of knowledge were successful or not. The last phase considers sustainability to ensure continuity of the intervention that has been shown to be effective.

With a population of 60.6 million (Stats SA, 2022) and approximately 84% of the population relying on public health services (National Treasury Fiscal Review, 2017), holistic patient care is a challenge in SA. One aspect of care often compromised is oral care, which falls within the remit of nurses. Given this statistics, public healthcare institutions in SA are stressed and overburdened, with those working in the system dealing with the ramifications (Khamisa et al., 2017; Manyisa & Van Aswegen, 2017). Nurses being frontline workers in hospitals shoulder the burden of inundated wards; insufficient nurses to cope with the numbers of patients accessing health services, poor to no recognition for nurse contribution to patient care and so forth. High workloads for nurses have culminated in emotional and psychological burnout (Rispel, 2015). Having to cope with high patient numbers, *non-essential* routines such as oral care are compromised. This has adverse implications for the patient as more studies continue to document the positive outcomes of oral care for atrial fibrillation and heart failure (Chang et al., 2020), a lower incidence of pneumonia among frail individuals (Manger et al., 2017) and improved oral health with type 2 diabetes mellitus (Simon et al., 2019). A study of nurses working in intensive care unit (ICU) in SA found that despite knowledge of aspiration and contaminated secretions, nurses did not complete oral care (Perrie & Scribante, 2011). Participants reported seeing no evidence of the benefit of oral care to patient outcome. Oral hygiene when compared with other nursing routines is least enjoyed by nurses (Hanne et al., 2012). Medication dispensing, monitoring of vital signs and feeding are understandably considered priority nursing routines. This article contends that it is equally important for nurses to be equipped with the necessary knowledge and clinical skills to perform oral care efficiently given its documented contribution to patient well-being and recovery (Hanne et al., 2012). Considering the context described, it appeared that the K2A model could offer a solution to address the needs of the context and the implementation of oral hygiene and oral care by nurses. This was explored in this study.

## Research methods and design

The aim was to explore the use of the K2A model to facilitate knowledge translation in oral care among nurses working in an acute general medical ward in SA. A qualitative design with ethnography was employed. Ethnography provided direct access to what nurses did, as well as what they say they did. Oral care in terms of frequency and the routine was observed as part of the ethnography. Ethnography including video-recordings, semi-structured and informal interviews were the tools used in the study. The research was undertaken within the medical wards in an acute, adult intake government hospital in Johannesburg, SA. A total of 139 nurses were recruited using random purposive sampling. Nurses from different categories as detailed here, were included as participants (Subedar, 2005):

- Enrolled nursing auxiliary or assistant nurse: A 1-year programme at college or exited after completing the first year of the university 4-year programme and competent to practice elementary nursing.
- Enrolled nurse: A 2-year programme at a nursing college or exited after completing 2 years of the 4-year-programme at university and competent to practice basic nursing.
- Professional or registered nurse: A 4-year-programme at university or a nursing college and competent to practice comprehensive nursing and midwifery.

All participants provided written consent to participate in the study. Consent from hospital management was obtained and was followed by ethnography of the medical wards over a 5-month period (January 2013 – end May 2013) and proceeded with once weekly in January 2013 – April 2013 to daily 6-h daily observations. The aim and content of the observations did not change. Any activity related to oral care was observed. Following training, the oral care routine itself that used by nurse participants was observed in terms of how it was performed, utensils used, time it took, and level of participation from the patient. Over the weeks, changes in these aspects were observed. A needs analysis discussion with nurses followed the ethnography. A pilot study was conducted (described below) followed by data collection for the main study (which involved training and monitoring of oral care implementation). Data were analysed using inductive thematic analysis. Analysis was data-driven to avoid bias given the volumes of published work (nationally and internationally) that provide a negative and unfavourable view on nurses, nursing culture, and nursing as a profession (Aikman, 2019; Crawford et al., 2019; Drennan & Ross, 2019). Data from the ethnography, semi-structured and informal interviews as well as video and audio recordings (of nurse training, nurse implementation of oral care and at mealtimes) were compared and checked against each other. The video and audio recordings were conducted before, during and after mealtimes, but with no set schedule. Flexibility in terms of when recordings were completed was necessary to accommodate the nurses and how they worked. All video recordings were completed by the author. The oral care routine was reviewed and completed using a checklist (Appendix 1). Respondent validation occurred during the course of the interviews. The oral care protocol was self-developed as a synthesis of existing standard hospital oral care protocols (in SA) and that developed by Brady et al. (2006). Frequency of oral care provision, use of equipment (toothbrush, oral suctioning unit), and resources (toothpaste, mouth rinse, lip care, dental floss) were observed.

### Ethical considerations

Ethical clearance to conduct this study was obtained from the University of the Witwatersrand Human Research Ethics Committee (No. M091166).

## Results

Literature concedes that process, context and sustainability are core components of any intervention study such as the one reported in this article (Grimshaw et al., 2012; Pinnock et al., 2017; Shepperd et al., 2009). Pinnock et al. (2017) highlight the importance of the reporting format and content thereof to ensure that effects of the intervention can be reasonably translated into clinical practice for the benefit of the individuals from that context. The results cover these aspects commencing with identification of the practice gap to implementation and finally sustainability of the intervention as per the K2A model.

***Problem identification – The gap in practice (Figure 1)*:** The ethnography provided the researcher with an overview of oral care implementation in wards. Inconsistent to no oral care before breakfast and no rinsing of mouth after any meal was observed. Independent patient tooth-brushing was observed for a minority of patients with toothbrushes. The observations were unpacked in a needs analysis discussion with hospital management, the senior nurses, that is, nurse sister, and the nurses caring directly for patients. All stakeholders acknowledged oral care as a low priority routine (the gap in practice), resulting in poor to no implementation thereof.

***Contextual needs – Adapt knowledge to local context (Figure 1)*:** The ethnography and needs analysis discussion concluded the following:

- Less than ideal working conditions for nurses.
- Limitations in inter-professional communication between nurses and medical and allied health professionals.
- A gap in oral care for patients.

A consensus was reached on the need for oral care training for nurses. The format and structure of the training were explored and confirmed as part of the pilot study, taking into account the variables within the context (i.e., number of nurses in a ward at any given time, nurse to patient ratio, and time availability).

Pilot study (part of adapt knowledge to local context): The pilot study aimed to confirm the length of the training session, the content validity of the information, and that the materials to be used for training were appropriate. The training format and structure were also confirmed. Altering the context to suit the needs of the study was not considered as per the K2A model. Training had to support the context. Results of the pilot study confirmed that one-on-one training at the patient bedside was most feasible as:

- there was less need for planning, preparation, and coordination
- there was no need to consider logistical variables such as venue, time for training, or audience participation
- the one-on-one model facilitated understanding with increased opportunity to address concerns and provide clarification
- it guaranteed hands-on experience by participant with opportunity for constructive feedback
- it did not impact nursing routine or compromise service delivery to any patient from a time perspective.

This training format addressed the requirements of the context, and was acceptable to hospital managers, nurse managers, and nurses. The materials used in the training, that is, the posters, booklet on oral care and pictures (all developed by the author specifically for the intervention) as well as the resources used, that is, gauze, forceps and mouthwash solution, were acceptable to the nurses and available within the wards. Adapting to the needs of the context supported the K2A model and contributed towards sustainability of the intervention, that is, oral care training. It also facilitated capacity building for nurses with maintenance of skills for use with all patients after completion of the study. Following consent from the patient and nurse, training was conducted at the patient bedside. The trained nurse subsequently implemented oral care (Appendix 1: Oral care protocol) until patient’s discharge.

The K2A model acknowledged the continuous movement and interaction between knowledge creation and the surrounding action cycle, highlighting that knowledge is continuously changing and that this in turn required action (practice) to change accordingly. The information and tools were therefore purposefully selected to ensure that they would be acceptable and useful to nurses to facilitate change in practice (Ilott et al., 2013b).

***Adapting knowledge and assessing barriers to knowledge use (Figure 1)***: In view of the context of oral care in the hospital described here, careful consideration of barriers and facilitators to implementation and involvement of the nurses was critical. To enhance inclusivity, nurse managers, nursing sisters, and nurses were involved in the design, content and delivery of the training to prevent disconnection between the drivers of the intervention and those implementing the intervention (Gruen et al., 2008). Barriers that emerged from the ethnography included:

- Poor to lack of monitoring of routines carried out by nurses:

‘Sad to say but she [*indicating nursing Sister*] doesn’t even know what I do for sure …’ (Participant 23, female, enrolled nurse)

- Time-consuming routines.
- Unknown benefit for nurses.

‘… doctors act like they only should be thanked for getting the patient better. What do we do? [*pause*] nothing?’ (Participant 51, female, enrolled nurse)

- Attitude and behaviour.

Modelling and demonstration, direct one-on-one feedback and individually tailoring the level of information provision was implemented to accommodate the different levels of qualification of the nurse participants. Adopting an interactive and participatory model as opposed to a didactic model of instruction produced favourable nurse participation and transfer of clinical skills (Tryssenaar & Gray, 2004). Provision of a continuing professional development certificate to recognise the training on oral care and opportunity for professional responsibility facilitated this process.

***Select, tailor, and implement interventions (Figure 1)*:** Oral care training was collaborative involving the nurse participants, researcher, as well as nurse and hospital management. There was consensus in the design of how the training would happen, the content of the training, the duration and manner of training. Based on the barriers identified here, the aids to training were selected. Table 1 provides a list of supplementary materials used.

**TABLE 1:** Supplementary material used during training.

Material and resources used	Description
Written aid in the form of a booklet.	This contained information on dysphagia and oral care, the procedure and resources required for oral care, explanation and rationales for protocols, problem-solving recommendations as well as frequently asked questions and definitions.
Flow-charts, signs and posters above patient’s beds.	This provided easy to refer to tips and guidelines for nurses during feeding at mealtimes and when performing oral care for patients.
Training was completed at the patient’s bedside in the form of individual one-on-one training.	This allowed opportunity for direct modelling and monitoring, with the author first demonstrating how to, followed by the participant. Modelling was preceded by a general discussion on dysphagia, and how oral care is linked to it, with emphasis on patient prognosis, aspiration and aspiration pneumonia.
Resources were kept to a minimum.	Oral care kits, suctioning equipment, and mouth wash were sourced from the ward.
The participant received a continuing professional development certificate on completion of the training.	This addressed the concern of attitude and provided an incentive for the participant.

The involvement and collaboration with all stakeholders aligned with requirements of the K2A model (Graham et al., 2006).

***Monitor knowledge use (Figure 1)*:** All nurse participants recorded each oral care session that was completed. Oral care was completed in the morning before breakfast; mouth rinsing after breakfast, lunch and dinner; and mouth rinsing had to precede any consumption of water (Appendix 1). Furthermore, nurses were required to record each completed mouth care session. Recordings were reviewed daily and incomplete records were brought to the attention of the nurse participant. Randomly timed video recordings of the nurses completing the oral care routine were captured. Cross checking the written records, the video recordings and information from the interviews completed the triad of data collection sources. The use of multiple sources allowed verification of information from one source against information from a different source. Recordings indicated that oral care was implemented 99.7% of the time. Three ‘no recordings’ were identified. Table 2 captures some comments from the nurses under their recording of mouth care.

**TABLE 2:** Examples of nurse’s notes for mouth care.

Difficulties	Successes
‘Unable to spit. Had to use suctioning’ ‘Mouth bleeding’ ‘Unable to open mouth. Teeth clenched. Had to force open’ ‘Patient not cooperative’ ‘Unable to clean tongue well. Patient refusing’	‘Patient cooperative’ ‘Patient able to move tongue around and spit out’ ‘Successful – patient cooperative’ ‘Able to complete suctioning successfully – patient tolerated well’

Note: Time, priority of other nursing routines and workload contributed to incomplete recording.

‘I was very busy because I still had to give the other patients their food …’ (Participant 29, female, assistant nurse)

‘I was very busy yesterday. I was rushing …’ (Participant 83, female, enrolled nurse)

Video recordings provided evidence that nurses were correctly implementing oral care in line with the training and schedule. The protocol presented with some challenges for patients who required suctioning largely because of contextual shortcomings where suction equipment at the patient’s bedside was not always in working condition. However, when the suction equipment was working, oral care for the patient requiring suctioning was in fact shorter by 3 min – 4 min on average. The video recordings confirmed that less patient compliance and cooperation was needed as the patient was not required to spit out or rinse their mouth. The nurse was able to accomplish this using the suctioning equipment.

***Evaluate outcomes (Figure 1)*:** The outcomes of the study aligned with the following objectives that were achieved: (1) feasible and accepted training method and strategies for knowledge transfer of oral care, (2) regular implementation of oral care and (3) sustained implementation of oral care. As observed, one-on-one training was cost-effective, time-effective, and enhanced knowledge translation of the content. The outcome of the training was positive. Themes identified from the interviews were:

- Learning opportunity

‘I am glad I got to learn the procedure for mouth care. I can see it is important and it is helping the patients’. (Participant 3, female, assistant nurse)

‘Mouth care is a new thing that I learnt. I can now do with all my patients. I can see how it is helping Mrs Y, so I will continue’. (Participant 11, male, nurse assistant)

- Improved patient care and service provision

‘The training for the mouth care was really good. I learnt something new and now I can use it for other patients …’ (Participant 27, female, nurse assistant).

- Opportunity for certification

‘I love it. The training we got now was lovely and to get a certificate to show that you have learnt it and are doing the mouth care correctly is lovely. It’s also good for my CV because other people can see exactly what you have been trained in’. (Participant 62, male, nurse assistant)

- Improved knowledge of other professions

‘I think the training and working together goes together. I’ve seen how by working together I understand better what’s happening with the patient and why certain things need to be done. I am sure it is the same for you. I think it is important to work together’. (Participant 14, female, enrolled nurse)

- Mouth care: It was practical, facilitated knowledge translation to other patients, and patient improvement was noted.

Recordings of oral care were reviewed for consistency, completion, and content.

***Facilitating sustainability of the intervention (Figure 1)*:** Sustainability was ensured via these measures:

- The research assistant was a speech pathologist and obtained a permanent position of employment at the research site, where she remained for 5 years after the completion of the study. This facilitated *institutionalising* of oral care.
- The nurse manager within each medical ward supplied the newly appointed speech pathologist who was previously the research assistant, with a list of the new nurses rotating through the ward and who required training on oral care. Institutionalising of the training was the goal.
- A review of training records 1-year post-completion of the study indicated regular weekly training sessions (on average eight training sessions per week). These training sessions were offered by the research assistant or speech pathologist on an individual basis (as per the study), to any new nurse or nurse from any ward who approached her for training. The oral care provision therefore expanded and was implemented to any in-patient in the hospital by the nurse who received training and was caring for that patient. While training was initially to nurses in the wards that the research assistant or speech pathologist worked as part of her patient management, knowledge and awareness of the training spread by word-of-mouth, hence nurses from wards with patients not requiring speech pathology intervention approached the research assistant or speech pathologist for training.
- Presentation of the findings of the study to the hospital and nursing management at the end of the study and a report on the continued training on oral care was provided 1-year post-completion of the study.

## Discussion

This study confirmed the value of extensive stakeholder consultation to alleviate misfit between stakeholder and facility needs and implementation of intervention strategies (Wensing & Grol, 2019). Macleod et al. (2014) elaborated on the financial, patient and societal ramifications of failure to act on research findings. It was against this backdrop and in considering the harmful consequences of poor knowledge translation, specifically in the area of oral care that this study was undertaken.

The study highlighted the following barriers:

- barriers between staff from different professions (e.g. limited communication and information exchange between nurse and speech pathologist impacting service delivery)
- systemic barriers (e.g. poor monitoring of nurse services by nurses perceived as a lack of interest)
- organisational and structural barriers (e.g. a lack of incentive and poor acknowledgement of nurse contribution to patient care).

Similar barriers, that is, professional, systemic and organisational were noticed by Grimshaw et al. (2012). Thus, the intervention was tailored to address these barriers. Consultation, active engagement, and hands-on involvement from all the listed stakeholders at each step of the research process promoted more communication exchange, patient discussion, and consultation. The value of health managers, health administrators and on the ground healthcare practitioners in the conceptualisation, design and implementation of interventions was clear (Morsiani et al., 2017; Wensing & Grol, 2019). The K2A model addressed the issue of a lack of monitoring by management and consequent feelings of disregard felt by the nurses. The buy-in, understanding and commitment from managers and nurses from the conceptualisation of the study to the implementation was key to the success of the intervention.

Interlinked to the context, was behaviour and attitude. Typically, professionals work in silos albeit for the same patient. As noticed here, high patient numbers and poor working conditions adversely impacted work morale and job satisfaction among nurses in SA (Mayosi & Benatar, 2014; Nesengani et al., 2019). Behaviour and attitude are recognised as two of the greatest challenges in knowledge translation historically (Mallidou et al., 2018). The training platform prescribed to a collaborative model of patient care that required frequent and direct communication between the nurse and other health professionals. Implications of holistic patient care are implicit within a collaborative model, and this was attained in this study. Knowledge translation to address a knowledge-to-action gap requires active collaboration between the researcher and knowledge-users and when achieved, the benefits of knowledge translation are results that extend beyond the lifetime of the research project. There was evidence to support that oral care for patients continued after completion of the project. The K2A model offered the researcher clear direction and directives on considerations for the project. It enabled use and practice of strategies and techniques in an efficient and effective manner thereby avoiding additional burden on already overstretched services and staff. Regarding the outcome of collaboration, a lesson learned was ensuring that the benefits of collaboration were not gained at the cost of further levels of stress and frustration for the nurse.

Effective communication enhanced implementation of oral care with provision of direct support, one-on-one training, and monitoring structures (Wensing & Grol, 2019). The study confirmed that a linear, unidirectional and passive flow of information from research to practice was incongruent with attaining knowledge translation as observed by Weiss (1979). The need for face-to-face contact with and involvement of the nurses in the process improved collaboration when caring for patients. The findings of the study echoed the sentiments of Cornwall and Jewkes (1995) ‘research strategies that emphasize participation are gaining greater respectability and attention within mainstream health research in developed and developing countries’. Nurse participation reinforced their active contribution to patient-related decisions and this in turn enhanced nurses’ autonomy.

This study was deemed successful in the sense that nurses implemented oral care with minimal prompting and evidence in the form of positive outcomes for the patient. The benefit of the study for nurses within the SA context was also made explicit by one nurse participant:

‘If we don’t push ourselves to learn new things, other people are going to take over and the nurses will continue to have a bad name. If we start and we show the doctors and the families that we know what we are doing and we care, then even the matrons will see it. We must all remember why we chose to do nursing’. (Participant 67, female, registered nurse)

## Conclusion

The K2A model provided the theoretical and clinical framework for the study as it facilitated information transfer on oral care among a group of nurses working in an acute government hospital in SA and enabled practical implementation of oral care by the nurses who received the training. This had relevance for SA and health professional researchers such as speech pathologists, given the healthcare challenges and resource constraints. The model was applicable to the needs of the study, the complexity of the context, and stakeholders. Importantly, it promoted adaptation of the required knowledge to suit the context and not vice versa. For contexts that offer unique challenges such as those faced by government healthcare institutions in SA, this was ideal and critical to the success of the intervention. In closing the existing gaps in clinical practice in SA, the importance of engagement and consultation with the end-users of the intervention cannot be over-emphasised. Edwards et al. (2019) emphasise the critical need to identify viable methods for knowledge translation in developing contexts such as Africa, to capitalise on shared-learning given that research funding, research initiatives and research capacity are often limited in these contexts. Efforts in research initiatives with appropriate theoretical frameworks may provide a platform to address gaps in practice and enhance patient care and inter-professional partnerships while managing patients.

While sustainability was tracked and monitored from a qualitative perspective, clearer quantitative measures were not completed. Thus, this component of the action cycle was acknowledged as a limitation of the study. Although measures such as journaling and debriefing were completed throughout the research process for the researcher, it is possible that elements of subjectivity resulted in a more favourable perspective of nursing by the researcher.

### Implications for nursing management

- Recognition of nursing staff for their role in patient recovery.
- Involvement of nursing staff in patient management decisions and interventions.
- Need for evidence to align with implementation of interventions.
- Implementation of research findings into clinical practice must consider context, nurse buy-in and input, and sustainability.

### Implications for healthcare professionals

- As the requirement for contextually relevant research to underpin clinical practice is increasingly expected in SA, with the systemic, financial and capacity challenges within healthcare, shared-learning offers an advantage. This study provides a theoretical framework that works in public healthcare and could be used to underpin future intervention studies by speech pathologists.
- Improvement in professional practice, patient service provision and patient outcomes continues to be challenging in developing contexts. Barriers to improvement and change are multifaceted. Understanding and involving the stakeholders, that is, staff and patients is an initial step in facilitating change that may be sustained.
- Empirical research to underpin speech therapy practice is necessary. However, when the parameters of research become too strict and exclusionary, implementation of research findings is compromised. Speech pathologists engaging in research in healthcare facilities must be cognisant of the balance between stakeholder and patient directed needs, rigour and integrity of findings, feasibility of interventions, as well as implementation of research findings.

## Appendix 1

### Oral care protocol and checklist

Patient Name/Number: ______________________________

Date: _____________________________________________

**Table:**

MORNING		MIDDAY		AFTERNOON		EVENING
□ 1. Weight	Water given on request or offered periodically	□ 1. Lunch (NO Water)	Water given on request or offered periodically	□ 1. Snack (NO Water)	Water given on request or offered periodically	□ 1. Supper (NO Water)
□ 2. Body Care		□ 2. Oral Care		□ 2. Oral Care		□ 2. Oral Care
□ 3. Oral Care		□ 3. Recording		□ 3. Recording		□ 3. Recording
□ 4. Breakfast (NO Water)						
□ 5. Oral Care						
□ 6. Recording						

**PLEASE NOTE:**

1. Oral care must be provided **before breakfast.**
2. The patient **must rinse out their mouth after every meal.** A visual inspection of the oral cavity must be completed by the nurse.
